# Comparative study between admission, orthopaedic surgery, and economic trends during Covid-19 and non-Covid-19 pandemic in an Italian tertiary hospital: a retrospective review

**DOI:** 10.1186/s13018-021-02754-2

**Published:** 2021-10-15

**Authors:** Gianluca Testa, Marco Sapienza, Fabrizia Rabuazzo, Annalisa Culmone, Fabiana Valenti, Andrea Vescio, Vito Pavone

**Affiliations:** Department of General Surgery and Medical Surgical Specialties, Section of Orthopaedics and Traumatology, A.O.U. Policlinico Rodolico – San Marco, University of Catania, Via Santa Sofia 78, 95123 Catania, Italy

**Keywords:** COVID-19 pandemic, Emergency room, Lockdown period, Orthopaedic hospitalisations, Surgeries, Economic trends

## Abstract

**Background:**

The COVID-19 pandemic represents one of the most massive health emergencies in the last century and has caused millions of deaths worldwide and a massive economic and social burden. The aim of this study was to evaluate how the COVID-19 pandemic—during the Italian lockdown period between 8 March and 4 May 2020—influenced orthopaedic access for traumatic events to the Emergency Department (ER).

**Methods:**

A retrospective review of the admission to the emergency room and the discharge of the trauma patients’ records was performed during the period between 8 March and 4 May 2020 (block in Italy), compared to the same period of the previous year (2019). Patients accesses, admissions, days of hospitalisation, frequency, fracture site, number and type of surgery, the time between admission and surgery, days of hospitalisation, and treatment cost according to the diagnosis-related group were collected. Chi-Square and ANOVA test were used to compare the groups.

**Results:**

No significant statistical difference was found for the number of emergency room visits and orthopaedic hospitalisations (*p* < 0.53) between the year 2019 (9.5%) and 2020 (10.81%). The total number of surgeries in 2019 was 119, while in 2020, this was just 48 (*p* < 0.48). A significant decrease in the mean cost of orthopaedic hospitalisations was detected in 2020 compared (261.431 euros, equal to − 52.07%) relative to the same period in 2019 (*p* = 0.005). Although all the surgical performances have suffered a major decline, the most frequent surgery in 2020 was intramedullary femoral nailing.

**Conclusion:**

We detected a decrease in traumatic occasions during the lockdown period, with a decrease in fractures in each district and a consequent decrease in the diagnosis-related group (DRG).

## Introduction

In December 2019, in the city of Wuhan, China, the first cases of acute respiratory syndrome from COVID-19 were identified, and the virus responsible for COVID-19 was later identified as SARS-COV-2 in February 2020. Due to the virus’s high contagion capacity, the World Health Organization (WHO) declared this a pandemic in March 2020 [1]. Clinical presentation of COVID-19 ranges from absence of symptoms to severe pneumonia [2] and could be associated to musculoskeletal symptoms, including myalgia, arthralgia, and fatigue, are a nearly constant presence [3]. COVID-19 has caused millions of deaths worldwide and was considered a massive economic and social burden. Social distancing was the first of the countermeasures employed by most countries. During the Italian national lockdown, there have been enormous restrictions on all daily living, work, and sports activities [4]. Similarly, considerable efforts have been made to contain the spread of the virus, and several safety measures have been adopted by hospitals and health workers to ensure the safety of the community [4]. The redistribution of hospital staff and resources in favour of those departments dedicated only to dealing with the health emergency led to the reduction or the complete cessation of elective medicine [5]. Furthermore, as a result of the measures adopted, the number, type, and methods of access to first aid centres and hospital emergency rooms have changed compared to previous years [6].

Although many hospital departments have suffered a significant contraction in their activity and even closure, our Orthopaedics and Trauma Department continued to work, reducing elective activity and leaving ample space for traumatology [4]. In order to avoid delay in trauma diagnosis and treatment, as well as, crowd the Emergency Departments (ED) [7], the several admission procedures has been proposed and published [8–10]. Despite the reduction of clinical activity was previously documented, no specific economic report are present in the literature. The purposes of this study were to (1) describe whether and how the COVID-19 pandemic has affected the need for orthopaedic intervention, the type of traumatic injury, and the characteristics of those patients who had first access for trauma to the ED during the lockdown period between 8 March and 4 May 2020; and (2) to compare the obtained results with those of the same period of 2019. We hypothesise that ER trauma admission was reduced in 2020 relative to the previous year.

## Materials and methods

### Design of the study

Between 8 March and 4 May 2020 (lockdown in Italy), a retrospective review of ER admission and discharges of trauma patients’ records was performed. A comparison was made to the same period of the previous year (2019). The study was conducted according to STROBE guidelines.

### Inclusion and exclusion criteria

Patients of any age affected by fractures that require orthopaedic surgery and hospitalisation at our operating unit, between 8 March and 4 May 2020, and the same period of the previous year were included. Cases of infection, pathological fractures, non-surgical patients, and elective interventions not compatible with access to the emergency room were excluded.

### Data collection

The 2019 and 2020 data of patients admitted to the Orthopedics Department of the Policlinico University Hospital, San Marco, was extracted from the ER database admission. For each patient who underwent surgery from 8 March to 5 May, gender, date of birth, date of exams, type of exams, diagnosis, report, type of surgery, date of surgery, and date of discharge were recorded.

We collected accesses, admissions, days of hospitalisation, frequency, fracture site, side, number and type of surgery, the time between admission and surgery, days of hospitalisation, and treatment cost, according to the diagnosis-related group (DRG). DRGs were created to assess resource utilisation and quality of care for hospital admissions (Table 1) [11].

**Table 1:** DRG codes, the cost of one day of hospitalisation, and the cost of each day of hospitalisation

DRG		Euro	First day of hospitalisation	Each day of hospitalisation
211	Intramedullary femoral nailing	6099	2050	176
219	Fibular fixation with plate and screw	4405	1887	209
544	Hemiarthroplasty of the hip	8837	0	205
234	Fixation of acetabulum fractures	4629	2296	243
219	Intramedullary tibial nailing	4405	1887	209
219	Fixation of humerus fractures	4405	1887	209
491	Shoulder arthroplasty	8565	0	230
224	Fixation of radius and ulna fracture	4391	1590	237
229	Fixation of carpus and metacarpal fracture	1266	1296	143
225	Fixation of tarsus and metatarsus fractures/lisfranc	2759	1684	217
219	EF of the elbow	4405	1887	209
224	EF of the forearm	4391	1590	237
211	EF of the femoral	6099	2050	176
219	EF of the leg/ankle	4405	1887	209
457	Fixation of clavicle fractures	457	61	49
229	Fixation of the hand phalanges fractures	1266	1296	143

*EF* external fixator

According to the patients’ age, the sample was divided into five groups (0–18, 18–40, 40–60, 60–80, over 80).

**Fig. 1:**
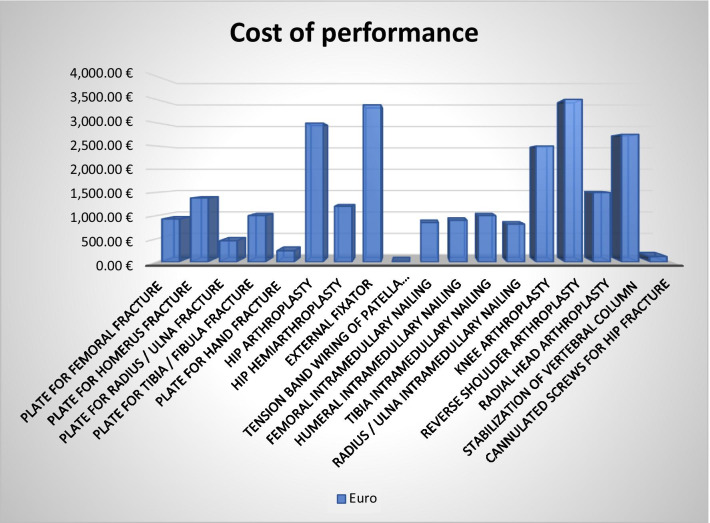
Cost of performance

Finally, the cost information for the performance was collected from the databases of the company’s health management, and the corresponding DRG is associated with each service (Fig. 1).

### Statistical analysis

Continuous data are presented as means and standard deviations, as appropriate. The chi-square test was used to verify the homogeneity of the two groups based on age, sex, and side of the lesion, while the analysis of variance (ANOVA test) was used to analyse two or more groups of data, comparing the variability within these groups with the variability between groups. The threshold selected for statistical significance was *p* < 0.05. All statistical analyses were performed using GraphPad 2016 software (GraphPad Inc, La Jolla, CA, USA).

## Results

**Fig. 2:**
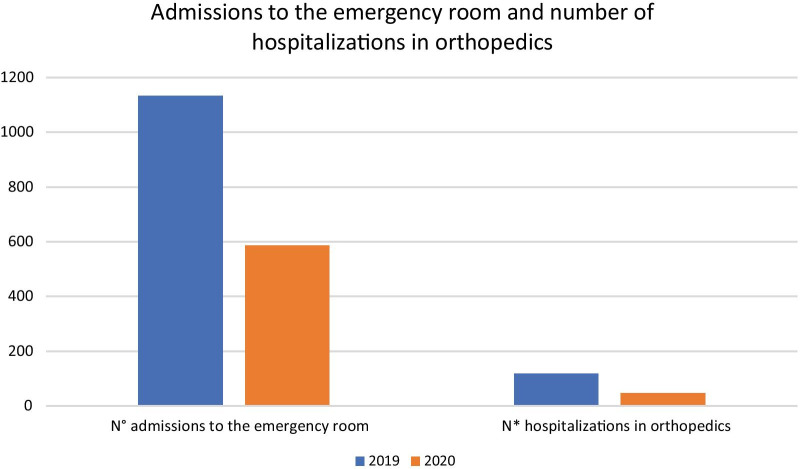
The number of emergency room visits and orthopedics admissions (*ER* emergency room)

In 2019 there were 1131 admissions to the emergency room for trauma, with 119 hospitalisations in orthopaedics (9.5%). In 2020, there were 519 admissions to the emergency room for trauma and osteoporosis, with 48 hospitalisations in orthopaedics (10.81%). No significant statistical difference was found for the number of emergency room visits and orthopaedics admissions (*p* = 0.53) (Fig. 2).

**Fig. 3:**
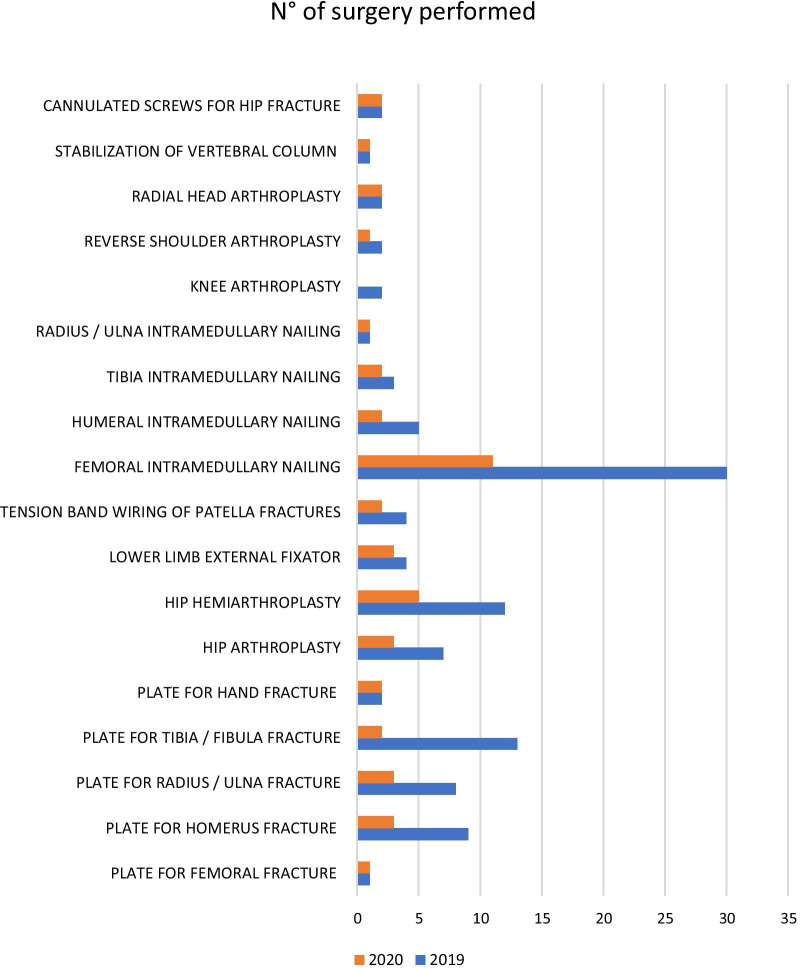
Difference in types of surgery in 2019 and 2020

The total number of surgeries in 2019 was 119, with only 48 in 2020 (*p* = 0.48). In 2020, admissions to the orthopaedics unit were 60.5% of the level recorded in 2019. All the surgical performances suffered a major decline in 2020, and the most frequent surgery was proximal femur intramedullary nailing in 2020. The data reveal a drastic reduction in proximal femur intramedullary nailing (− 91%), ankle fixation with plate (− 97.8%), and fixation of humerus with plate (− 77.6%) (Fig. 3).

High-energy trauma was reduced due to a reduction in high-energy traumatic occasions, but the number of home accidents involving the elderly was maintained.

**Fig. 4:**
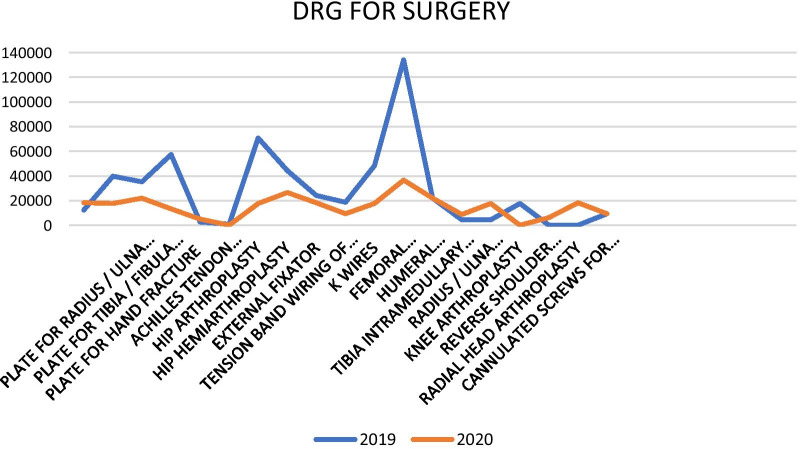
DRG for surgery

In the selected period of 2019, the Orthopedic Clinic produced a DRG of 545,479 euros (a mean of 28,709.42 ± 33,102.22 euros for each surgery); in 2020 it was recorded a decrease of − 261,431 euros (545,479 in 2019 vs. 284,048 in 2020; − 52.1%). A statistical significance was found in the comparison of the means (*p* = 0.005) (Fig. 4).

**Fig. 5:**
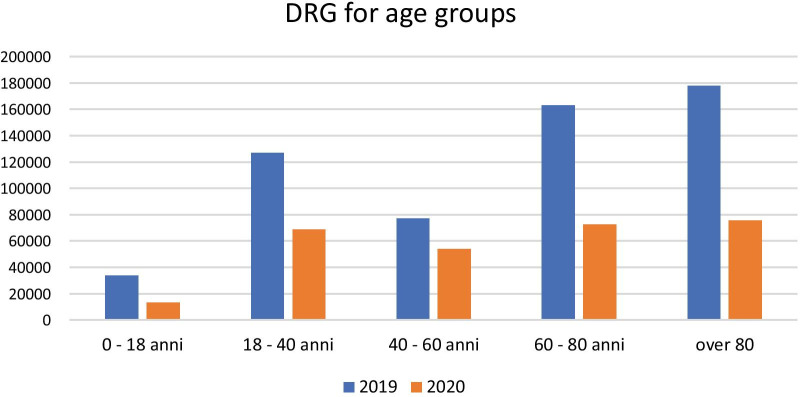
DRG for age groups

According to DRG by age group (Fig. 5), the Orthopedic Clinic recorded a substantial decrease in income (*p* = 0.0000), equivalent to a reduction of 20.289 euros for patients aged between 0 and 18 years (*p* = 1.0000), 58.161 euros for those between 19 and 40 (*p* = 1.0000), 23.178 euros for patients between 41 and 60 years (*p* = 1.0000), 90.594 euros between 61 and 80 years (*p* = 1.0000) and 102.411 euros for patients over 80 years (*p* = 0.0000).

**Fig. 6:**
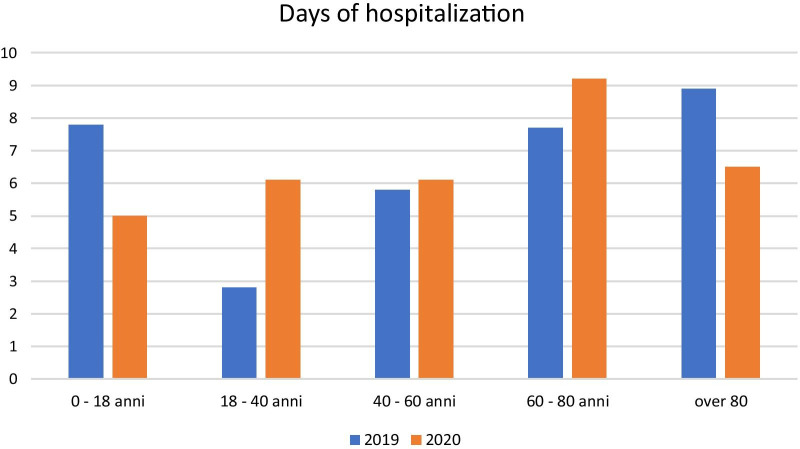
Days of hospitalisation for age group

The patients were divided by age group, and the days of hospitalisation were calculated for each group (Fig. 6). We detected a significant difference when comparing 2019 and 2020 (*p* = 0.05). In 2019, those patients up to 18 years and those over 80 had a longer hospital stay, and instead, in 2020, the 60–80-year-olds group had the most days of hospitalisation. The overall time of average hospitalisation and age were comparable between years (*p* > 0.05).

**Fig. 7:**
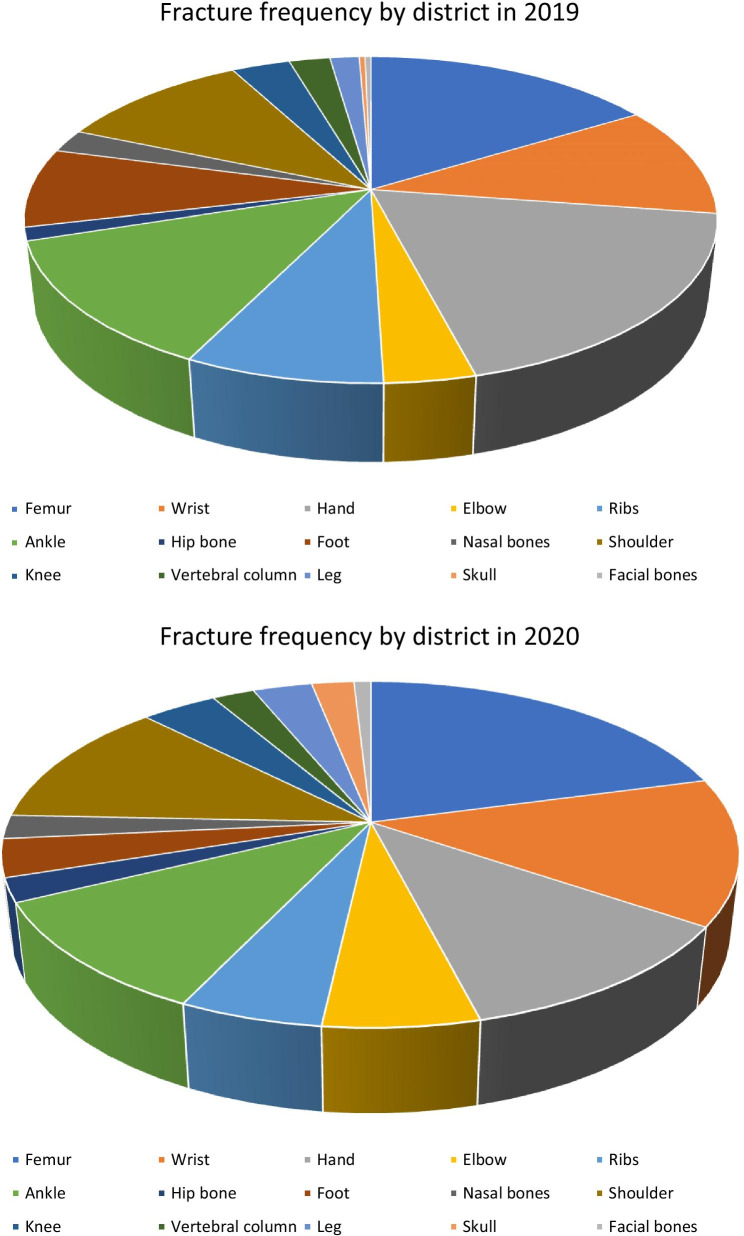
Fracture frequency by district in 2019 and 2020

A comparison was also made of the frequency of fractures by body districts (Fig. 7). There was a decrease in fractures in each district, and fractures of the femur, wrist (− 4.61%), hand (− 37.35%), shoulder (− 10%), and ankle (− 23.8%) were the most frequent injuries. 2020 saw a statistically significant reduction in the number of treated fractures of the femur (*p* = 0.0002), wrist (*p* = 0.006), elbow (*p* = 0.03), shoulder (*p* = 0.03), and leg (*p* = 0.09).

## Discussion

Since March 2020, the COVID-19 pandemic has profoundly influenced accesses to our Emergency Department, and a significant decrease of accesses (− 51.8%) has been recorded, with the number of hospitalisations in our unit decreasing by 60.5%.

Despite these decreases in the number of accesses to the emergency room and to our unit, no significant difference was found for the number of emergency room visits and orthopaedics admissions when comparing 2019 (9.5%) and 2020 (10.8%). Taking into consideration the DRG, there was a decrease for the chosen period of 261,431 euros, equal to − 52.07% between 2019 and 2020 (Fig. 5).

This trend could be justified considering the numerous indications provided by the government and the mass media, suggesting that people access the emergency room only for serious reasons that cannot be delayed in order to avoid contagion and the collapse of health facilities [9].

After the spread of coronavirus, governments of the world imposed restrictions on population activity to limit the spread of COVID-19 [12]. The first Italian infectious outbreak was registered in February 2020 in Codogno, Lombardy [13]. Italy was the first country to report a case of coronavirus in Europe, and it was the country with the highest number of deaths in the world for several months during the beginning of the pandemic. On 30 January, the Italian government declared a state of emergency. Then, on 22 February, the Italian National Institute of Health identified certain municipalities in Lombardy and Veneto as so-called red zones, which were quarantined for 14 days [14].

However, due to the rapid spread of the virus, the government proceeded to close all schools, most commercial activities, and many factories, also forbidding all sports activities in northern Italy on 7 March. These measures were then extended to the whole country on 11 March 2020 [15]. Within just a few weeks, many changes were made to cope with the increasing need for medical care and intensive care units.

To deal with the epidemic, the orthopaedics and traumatology units and many hospital units in the national territory had to help as much as possible. In the initial phase of the outbreak, orthopaedic and trauma teams were allowed to continue performing surgeries for trauma and cancer patients. As a result of these preventive measures, at Humanitas Hospital in Rozzano (Mi), the number of hip and knee arthroplasties fell sharply from 706 in 2019 to 166 (76.5% less) in 2020. Regarding the indications for arthroplasty, 95.6% of the surgeries performed in 2019 were rated as non-urgent, and the percentage was similar in 2020 (95.8%). A total of seven arthroplasties classified as urgent were performed in 2020. The number of patients admitted to the rehabilitation unit after arthroplasty was 323 in 2019 (46% of all TJAs) and 45 in 2020 (27%) [16]. In a similar way, rehabilitation units for orthopaedic patients also had to conform to the needs related to the pandemic emergency. The Italian Society of Physical and Rehabilitative Medicine (SIMFER) considered it necessary to maintain the appropriate levels of activity in this field under such difficult circumstances, not only to provide adequate care for people in need of rehabilitation. Therefore, a summary document has been filled with recommendations to provide adequate care in addition to protecting patients and professionals and remembering that the first priority is to limit the spread of infection [17]. Similar prescriptions have also been implemented in other countries [18–21]. In Galeazzi Orthopaedic Institute ER in Milan, the analysis of this aspect during the first month of the pandemic (12 March to 12 April 2020) compared to the same period in 2019 demonstrated marked differences in length of emergency department stay, request for chest radiographs, discharge diagnosis, triage color-code at admission and discharge (white code: non-urgent patients; green code: urgent but non-critical patients; yellow code: fairly critical patients; red code: patients at danger of death), and emergency department arrival and discharge modalities [22]. Comparable data were reported by the survey carried out by the CIO (Italian Osteosynthesis Club), which collected data from different centres on the Italian territory and verified the frequency of trauma access to the emergency department in the first 6 weeks of lockdown. Data from the hospital register showed that the number of accesses to the emergency room in the first week increased by 17%, and from the following week, the activity started to decrease by 28% in the second week, to 60%, 57%, 66%, and 71% in the following weeks [23]. Sports injuries increased by 70% in the first week, then there was a progressive decrease by 51%, 84%, 96%, 98%, until 100%, as outdoor team or individual sports activities were eventually prohibited. Considering injuries at work, there was a 60% increase in the number of traumas in the first week and a progressive weekly reduction of 30%, 73%, 55%, 63%, and 72% thereafter. Domestic injuries increased by 15% in the first week and then decreased by 25%, 41%, 40%, 56%, and 41% in the last week considered [23]. Although there was a significant reduction compared to the previous year, the only traumatic events that remained constant during this period were femoral neck fractures in elderly patients. Also, closer analysis showed that the number of hospitalisations differed by age group, with working-age patients being more affected than the elderly, even though femur fractures appeared to be the main cause of hospitalisation [23]. A decrease in femoral neck fractures in the elderly (2020 vs. 2019) was recorded in Italy, although less than the decrease in fractures due to high-energy trauma, as they occur due to falls at home. The same trend was confirmed by the present study; in fact, all surgical performances suffered a sharp decline in 2020, but the most frequent intervention was the proximal femur intramedullary nailing. High-energy trauma was reduced due to the reduction of high-energy traumatic occasions, but home accidents involving older individuals were not reduced. The Emergency Department of the AOU, Policlinico G. Rodolico, San Marco of Catania, registers the largest number of trauma patients in the area every day. Beyond the AOU, the institution also welcomed patients destined for other Emergency Departments that, at the time, were instead dedicated to patients with suspected COVID-19. According to our estimates, between 8 March and 4 May 2019 and the same period in 2020, there was a 51.8% decrease in trauma admissions and a − 60.5% decrease in hospitalisations for traumatic events. Despite the admission reduction, no statistical differences were founded compare to the previous year (*p* = 0.53), it could be warranted of proper admission criteria during the two periods. These data could be modified if we considered the elective interventions that were not carried out in 2020.

Catania is one of the largest and most populous in Sicily, with a population of approximately 300,000 inhabitants, and considering the adjacent cities, it serves a catchment area of more than one million citizens [19]. There are three different public hospitals in which there are emergency room accesses and orthopaedics and traumatology units that perform thousands of care services and major and minor orthopaedic surgery operations every year.

In Ireland, considering a study carried out on three trauma centres in Dublin, with care volumes similar to those of Catania (159 high volume bed), the average was 65 cases overnight and 100 cases per day per week and more than 1500 primary and revision hip and knee arthroplasties per year [24]. The authors report a similar mean age of admission as in this study (55 years; range 17–92 years). Also, there was a statistically significant decrease in the number of patients who had surgery per day (− 39.8%, *p* value < 0.001) between the reference and containment periods and between the pre-containment and containment periods [19]. With regard to hand surgery, there was a significant decrease only between the reference and containment periods (− 23.6%, *p* = 0.029). Also, the authors reported a 28.7% decrease in the activity of traumatic surgery after social activity restriction. Looking at general traumatic surgery, there was a 37.8% decrease in activity after the statement of restraint measures [19]. The change in traumatic activity depends on the occupation of the population. The authors reported a 28.7% decrease in the activity of traumatic surgery after social activity restriction. Looking at general traumatic surgery, there was a 37.8% decrease in activity after the statement of restraint measures. The change in traumatic activity depends on the occupation of the population. The most significant decrease in general orthopaedic trauma compared to hand trauma, which might be explained by the decrease in traumatology of the elderly and/or the decrease of road traffic accidents [19]. With regard to the anatomical districts, in our study, it was found that during the lockdown period, the main areas of injury were: the femur (23%), distal radius (15%), shoulder (13%), and ankle (12%). In the Irish study, 32% of the fractures were in the ankle and foot, 28% in the hand and ankle, and 16% in the hip and femur [19]. The most salient data in our study show a drastic reduction in proximal femur intramedullary nailing (− 91%), ankle fixation with a plate (− 97.8%), and fixation of the humerus with a plate (− 77.6%). However, the femoral fractures represent the most frequent fracture site during the lockdown period in 2020 (Fig. 4).

Regarding the DRG of our institutions of the AOU, Policlinico G. Rodolico, San Marco, we detected a decrease of 52.1% in 2020 relative to 2019, equivalent to 261,431 euros (Fig. 5).

Hashmi et al. [25] estimated that the financial income of one of Pakistan’s leading orthopaedic centres decreased by more than 55%. In the USA, some authors have noted immediate economic effects and fallout from the COVID-19 crisis, similar to those of the Great Recession of 2008 [26]. A survey found that orthopaedic surgeons lost almost 30% of their retirement savings during the 2008 economic recession, with surgery and patient volume decreasing by 30.4% and 29.3%, respectively [27]. After the Great Recession, from 2009 to 2011, the decrease in hospital admissions by patients with commercial insurance resulted in an average loss of $3.7 million for a 300-bed hospital. At the end of the COVID-19 crisis, unemployment is expected to be up to three times that of the Great Recession. While the healthcare systems considered are very different, the authors described an economic decline similar to that seen in our analysis. Unfortunately, to date, there is no scientific data or evidence to measure the actual economic consequence in Italy for orthopaedic and trauma units. To our knowledge, this is the first Italian scientific study to quantify the welfare and economic change of a department of orthopaedics and traumatology in a populous Italian city. No previous study was published about the economic variation trend in OU of orthopaedics and traumatology; moreover, the Italian results reported in the literature are limited to the northern region of the country. This article is useful to provide a more extensive picture of the health system reaction to an unusual pandemic. The present findings could be helpful in the creation of future clinical-care and economical strategies. Telemedicine is of great interest for the future in orthopaedics and is considered safe and effective by several authors [28]. Telemedicine could be used in the postoperative follow-up of selected cases, in the follow-up of fractures, and also in paediatric cases [28]. Remote fracture diagnosis is successfully performed on an outpatient basis in many countries. Protocols and methods have been developed to standardise the virtual orthopaedic examination for common musculoskeletal conditions. Satisfaction with teleconsultations and cost-effectiveness of remote care orthopaedics has been good [29, 30]. It is believed that remote care will be extended in several hospitals around the world because of its enormous potential. This fact is explained by the natural development of technology and the change and obliteration of habits that have accelerated exponentially since the COVID-19 crisis.

This study is preliminary, and further investigations are needed to confirm our data through multicentre studies conducted in large organisations like ours. It certainly shows the immediate changes that the pandemic caused on the health system, decreasing performance and the consequent DRGs with important professional and economic losses.

Limits of the study are the retrospective study design, limited follow-up, the lack of patients clinical assessment and more extending economical analysis.

## Conclusion

The COVID-19 pandemic has profoundly influenced our National Health System, forcing us to reorganise paths and activities. In Catania City, the fear of being infected by COVID-19 has undoubtedly played a leading role in reducing access to the emergency room and admissions to the orthopaedics department of the Policlinico-San Marco. Our data confirm the decrease in traumatic occasions outdoors, while those occurring within the home environment remained unchanged. We also noticed a consequent reduction in DRGs and a decrease in fractures in each district. Further multicentric and multidisciplinary studies will be needed to confirm the findings from this study.

## Data Availability

All analysed data are included in this published article.
